# Lung Cancer Prevention Among Appalachian Kentucky Women: A Community-Engaged Mixed Method Study

**DOI:** 10.1177/08901171251346607

**Published:** 2025-05-27

**Authors:** Jessica R. Thompson, Nancy E. Schoenberg, Pamela C. Hull

**Affiliations:** 1 Department of Health Policy and Administration, College of Health and Human Development, 8082 The Pennsylvania State University, University Park, PA, USA; 2 Community Impact Office, Markey Cancer Center, 4530 University of Kentucky, Lexington, KY, USA; 3 Department of Behavioral Science, College of Medicine, 4530 University of Kentucky, Lexington, KY, USA

**Keywords:** participatory mixed methods, community-engaged research, Appalachian region, lung cancer prevention, women’s health

## Abstract

**Purpose:**

Appalachian Kentucky (KY) residents experience the highest lung cancer rates in the US with declines lagging among women; we sought to uncover barriers and facilitators to lung cancer prevention for Appalachian KY women and to identify community-specific interventions.

**Approach:**

We utilized concept mapping, a participatory mixed method, to generate consensus on perceived barriers and facilitators.

**Setting/Participants:**

We recruited 71 adult women from Appalachian KY counties.

**Method:**

After collecting online concept mapping data, we used multidimensional scaling to generate a point map of perceived similarities and hierarchical cluster analysis to create a thematic cluster map. We compared average cluster ratings across importance and feasibility. During focus group discussions, we shared concept maps to gather insights on intervention areas.

**Results:**

Participants listed 70 barriers and facilitators in 8 thematic clusters, including community-level, healthcare, and tobacco-related factors. Participants identified three intervention areas: 1) educational campaigns, including efforts directed toward youth, mothers, and those eligible for lung cancer screening; 2) policy, such as smoke-free laws, inclusion of vaping in existing policies, and advertisement bans; and 3) improving access to lung cancer screening.

**Conclusion:**

Our findings support multilevel interventions for lung cancer prevention, including improving awareness, local policy, and screening access for Appalachian KY women. This research contributes novel understanding of local and gender-specific barriers and informs future Appalachian lung cancer prevention studies.

Lung cancer is the leading cause of cancer-related death for women in the US, including the Appalachian region.^1^ Although lung cancer rates have declined steadily among men since the 1990s, such decreases have not been seen among women.^1^ Associated with elevated mortality, smoking rates in Appalachia remain among the highest in the country, including those among women.^2^ However, other factors must be considered to address risk among women, as approximately 20% of women diagnosed with lung cancer are lifelong non-smokers.^1^ Lung cancer has an overall poor prognosis,^3^ which emphasizes the need for both primary prevention (e.g., tobacco use and radon risk reduction) and secondary prevention for early-stage diagnosis through low-dose computed tomography (LDCT) scans, the only currently recommended form of screening.^4^ The continued high smoking rates, incidence, and mortality among Appalachian women suggest culturally targeted strategies are needed to reduce lung cancer risk.

Lung cancer risk among Appalachian women extends beyond the individual level and requires solutions that address social and community contexts. Known lung cancer risk factors fall across social-ecological levels. At an individual level, in addition to the behavioral risk factor of tobacco use, studies show women are more likely than men to develop non-small cell lung cancer, most commonly found in non-smokers^5,6^; Appalachian women are 3.5x more likely to develop this type of lung cancer compared to their national counterparts.^7^ Additional social and community-level factors are vital to consider. State-level tobacco policies,^8,9^ insurance access,^10^ environmental exposures (e.g., second-hand smoke, radon),^11-15^ and historical livelihoods (e.g., farming, mining) have been connected to lung cancer risk,^3^ and psychosocial factors like chronic stress (e.g., caregiving stress) disproportionately affect women, contributing to adverse coping behaviors like smoking.^16^ Noted interactions also exist; for example, while radon exposure is dangerous, its harmful effects increase when combined with exposure to tobacco smoke.^17,18^

The socioeconomic inequities women in Appalachia experience support the need for risk reduction and prevention solutions capable of addressing multilevel factors. Overall, women experience higher poverty rates in the US than men.^19^ These socioeconomic inequities, which are more pronounced in Appalachia, have been tied to cancer among women, including increased behavioral risk factors, reduced access to preventive screening, and increased mortality.^3,20,21^ Appalachian women also experience healthcare access challenges (e.g., 39% of Appalachian counties lack the minimum number of needed primary care providers, who are the first line for preventive services, and 20% of Appalachian counties do not have a hospital).^22^ In part driven by these inequities, Appalachian women show low completion of preventive screening and follow-up referrals on risk factors like smoking cessation and nutrition.^23^ More research is needed to explore how to address the high levels of risk women in this region experience and to find preventive solutions that incorporate social, community, and environmental contexts.

## Purpose

Despite the high prevalence of lung cancer mortality among women in the Appalachian region, we currently lack published research on lung cancer primary or secondary prevention in this population.^24^ Particularly as a new and novel focus, community-engaged approaches provide an ideal way to capture local context and lived experiences.^25^ This study utilizes concept mapping, which is a community-engaged mixed method, to: 1) uncover the range of perceived barriers and facilitators to lung cancer primary and secondary prevention and 2) identify community-specific intervention ideas to prevent lung cancer among Appalachian women.

## Methods

### Design

We utilized an observational design with concept mapping methodology. As a community-engaged research method, concept mapping has been applied across a variety of health topics,^25,26^ including cancer-specific studies.^27-29^ Concept mapping is a participatory mixed method designed to provide structure, through quantitative activities, to qualitative data collection in order to reach group consensus on a particular question of interest.^30^ Within the realm of participatory approaches, concept mapping has the unique ability to prioritize wide-ranging factors and to generate community-specific action steps.^26^ The method follows six specific steps to generate conceptual maps in an iterative process: 1) Preparation, 2) Generation, 3) Structuring, 4) Representation, 5) Interpretation, and 6) Utilization.^30^ Three steps contain distinct data collection points, which include: brainstorming a list of items based on a focal prompt (*Step 2*), sorting and rating the resulting item list (*Step 3*), and providing qualitative feedback through focus group discussions in response to the generated concept maps (*Step 5*).

### Sample

From September to December 2022, we recruited 71 Appalachian Kentucky (KY) women to participate in concept mapping activities. Due to our interest in both primary and secondary prevention, individuals had to be adults (18+), identify as women, and have lived in one of the 54 Appalachian KY counties for at least 2 years to be eligible to participate. We recruited participants through: 1) existing lists of previous research participants interested in additional studies maintained by the study team; 2) ResearchMatch, a national electronic database and web-based recruitment tool^31^; and 3) the University of Kentucky Wellness Health & You (WHY) registry maintained by the University of Kentucky Center for Clinical and Translational Science.^32^ We distributed flyers and recruitment messages to each of these groups electronically, which included a QR code connected to an eligibility screener in REDCap.^33^ If potential participants who completed the screener met eligibility criteria, study staff contacted these individuals to provide study consent and log-in information for the concept mapping activities. Participants received up to $60 in gift cards ($40 for the online concept mapping and $20 for the group discussions). We received a waiver to document consent for this project. All activities were approved by the University of Kentucky IRB as expedited under protocol #78464.

### Measures

All online concept mapping activities for this project were conducted using the Groupwisdom platform developed by Concept Systems Inc.^34^ and Zoom video conferencing.^35^ The lead author, in consultation with the authorship team, developed the data collection components and timeline (*Step 1*); the lead author met with this team throughout the project to discuss progress and problem-solve any issues. The authors utilized results from the recent University of Kentucky Cancer Center KY Community Cancer Needs Assessment in the development of project content to ground the activities in community needs and feedback.^36,37^ Each of the Groupwisdom activities remained open for participants for 3-4 weeks, allowing individuals to log-in and contribute responses as feasible.

In the brainstorming activity (*Step 2*), we asked participants to respond to the focal prompt: *What are all the factors (good or bad) that affect the lung health of women in your community*? The authorship team cleaned the resulting list, including removing duplicates, which resulted in a final list of 70 unique items (Supplemental Table 1). Next, we asked participants to complete the sorting and rating activities (*Step 3*), in which participants group the identified items into groups that make sense to them and give each pile a thematic name. Participants then rated each item on two Likert-type scales: 1) *how important is this item for behaviors to keep your lungs healthy* (e.g., not smoking, getting lung cancer screening for those eligible) and 2) *how feasible would it be to address this item for women in your community*? Each scale had five-point response options, where 1 indicated not at all important/feasible and 5 was extremely important/feasible. Typical for concept mapping studies, we also collected a brief demographic questionnaire at this step, including age, race, ethnicity, educational attainment, and health insurance status for potential use in comparison of rating responses by important demographic characteristics.^30^

### Analysis

With the data resulting from the sorting and rating activities, we generated various conceptual maps and rating comparisons (*Step 4*). First, we utilized similarity matrices and non-metric multidimensional scaling to produce a point map from the sorting data, in which items with higher perceived similarity have reduced relative distance between them.^30^ Then, using hierarchical cluster analysis, we created a cluster map that depicts overarching thematic categories by grouping the points into clusters of highest perceived similarity. We combined the point and cluster map together for ease of interpretation by the participants. Using the rating data, we compared average cluster ratings across the rating scales with correlational values (r). We also used a Go-Zone plot, which utilizes bi-variate comparisons across the two rating scales, to compare item ratings within the highly rated clusters.^30^

We then brought these maps and rating comparisons to participants, with the goal of soliciting their interpretation (*Step 5*). We conducted three focus group discussions on Zoom, each of which lasted approximately 60 min and followed a semi-structured discussion of produced maps and figures. These discussions specifically focused on soliciting insights on items within the most highly rated clusters and on identifying potential intervention opportunities to address these items. All of the discussion groups were recorded and transcribed. We utilized the transcripts to identify representative quotes from the discussion groups to bolster the interpretation of the concept mapping findings.

## Results

### Participant Characteristics

Participants in this study (N = 71) had an average age of 48.5 years old. Approximately 28.1% of participants had less than a college education. Participants had diverse health insurance coverage, including over 50% with a public health insurance plan. Three-quarters of our sample identified as non-Hispanic white, which is slightly more diverse than the Appalachian KY region. Notably, we spoke to women in 34 of the 54 Appalachian counties in the state. Table 1 shows the descriptive demographics for the study sample.

**Table 1.** Demographics for the Concept Mapping Participants (N = 71).

Variable	Mean (SD) freq. (%)
Age	48.5 (15.8)
Educational attainment	
Completed high school or GED	5 (7.0)
Some college or vocational school	15 (21.1)
Bachelor’s degree or higher	51 (71.8)
Insurance type^a^	
Employer, military, or private	35 (47.3)
Medicaid	9 (12.2)
Medicare	28 (37.8)
Kentucky health insurance marketplace	2 (2.7)
Race/ethnicity^a^	
Black or African American	13 (17.1)
White	57 (75.0)
Hispanic, Latin American, or Spanish origin	4 (5.2)
American Indian or Alaska native	2 (2.6)

^a^Responses are not mutually exclusive.

### Cluster Maps and Names

**Figure 1.**
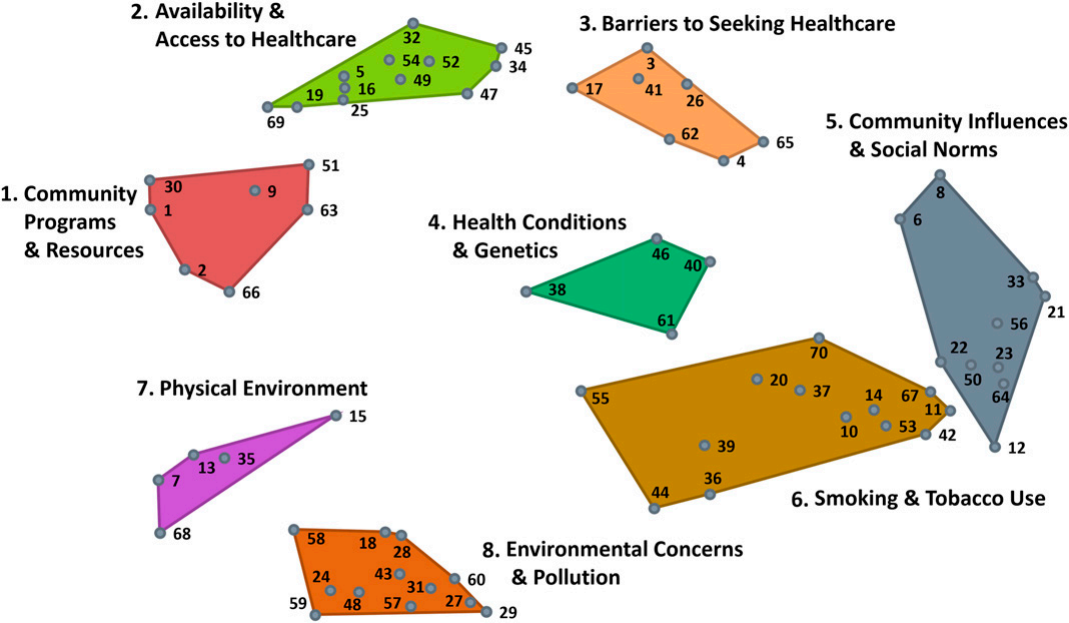
Combined Point and Cluster Maps From Sorting and Rating Data With Participant Developed Cluster Names.

The sorting and rating data from the concept mapping activities resulted in an 8-cluster solution. A list of all the items sorted by cluster is available in Table 2, and Figure 1 depicts the combined point and cluster map for the 70 unique items. The cluster names came from participant recommendations made during the pile sorting process, which were finalized during the interpretation discussions. The clusters include: 1) Community Programs & Resources, 2) Availability & Access to Healthcare, 3) Barriers to Seeking Healthcare, 4) Health Conditions & Genetics, 5) Community Influences & Social Norms, 6) Smoking & Tobacco Use, 7) Physical Environment, and 8) Environmental Concerns & Pollution.

**Table 2.** List of Final of Concept Mapping Brainstormed Items Sorted by Cluster.

Cluster	#	Item
1. Community programs & resources	1	School programs about lung issues and the negatives of smoking
	2	Availability of healthy and affordable foods
	9	Education on lung health and the side effects of smoking
	30	Education/programs on the importance of lung cancer screening
	51	Exposure to good health practices/behaviors
	63	Maintaining lung health as a top priority by those in power
	66	Access to exercise facilities (e.g., walking tracks, gyms, local parks, etc.)
2. Availability & access to healthcare	5	Local health facilities with lung screening and treatment options
	16	Free healthcare and screening to detect lung health issues early
	19	Use of prescription inhalers and medicines to improve lung health
	25	Ability to get screened at a younger age
	32	Access to transportation to doctor’s appointments
	34	Distance to treatment facilities
	45	Cost of lung care/treatment services
	47	Quality of available healthcare
	49	Having a primary care doctor
	52	Availability of healthcare providers
	54	Medical facilities for the healthcare of women
	69	Training for health care workers on screening and detecting lung cancer
3. Barriers to seeking healthcare	3	Fear they won’t be able to afford doctor appointments or treatment
	4	Not attending to their own health to take care of children or partners
	17	Getting/staying healthy is too expensive
	26	Fear to go to the doctor because of what the diagnosis may be
	41	Not going to the doctor until the symptoms are severe
	62	Low household income
	65	Untreated medical conditions
4. Health conditions & genetics	38	High asthma rates
	40	Obesity
	46	Health conditions that can make lung problems worse
	61	Genetics
5. Community influences & social norms	6	Stress women experience in their lives
	8	Attitude of “it is what it is” when it comes to health
	12	Alcohol use/excess drinking
	21	Not caring what others think/social pressures about quitting smoking
	22	Use of smoking among people in recovery/treatment from drug abuse and alcoholism
5. Community influences & social norms cont	23	Acceptance and normalization of smoking
	33	Peer pressure
	50	Substance use
	56	Growing up watching others, including women, in the family smoke
	64	Smoking as a way to socialize
6. Smoking & tobacco use	10	Availability of tobacco, vaping, and nicotine products
	11	People smoking at a young age
	14	High smoking rates
	20	Side effects of smoking
	36	Allowing smoking in public spaces
	37	Effects of overdose drugs (e.g., naloxone)
	39	Growing up around second-hand smoke
	42	Acceptance of vaping, including indoors
	44	Second-hand smoke in the community
	53	High vaping rates
	55	Advertisements aimed at young people
	67	Co-use of smoking with other drugs and alcohol
	70	Mothers smoking while pregnant
7. Physical environment	7	Fresh air
	13	Older homes and structures with expensive upkeep
	15	County smoke-free policies for not smoking in buildings
	35	Availability of safe drinking water
	68	Large and established forests
8. Environmental concerns & pollution	18	Air fresheners
	24	Climate conditions
	27	Chemicals/pesticides being used in local communities
	28	Working in places that could be risky to lung health
	29	Pollution from vehicles (e.g., cars, diesel trucks, farm machinery)
	31	Environmental toxins in the home like plastics and radon gas
	43	Asbestos in houses and other buildings
	48	Run-off from coal mines
	57	Pollution from manufacturing companies, plants, or factories
	58	Pollen in allergy seasons (spring and fall)
	59	Open burns of vegetation and garbage
	60	Miners coming home covered in coal dust

### Cluster Ratings

**Figure 2.**
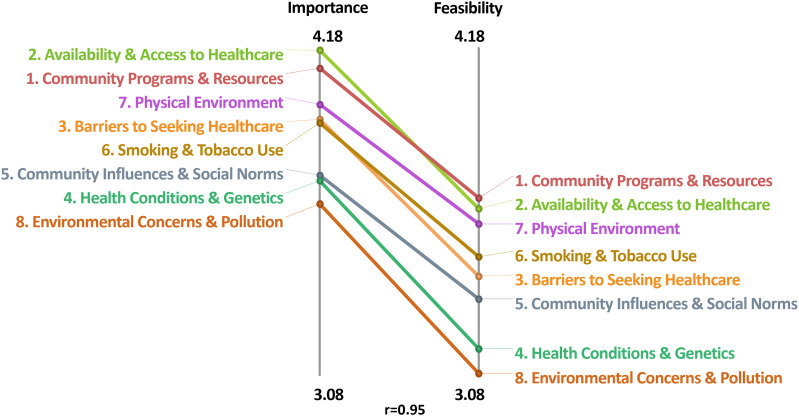
Comparison of Average Cluster Ratings Across Rating Scales (Importance and Feasibility).

**Figure 3.**
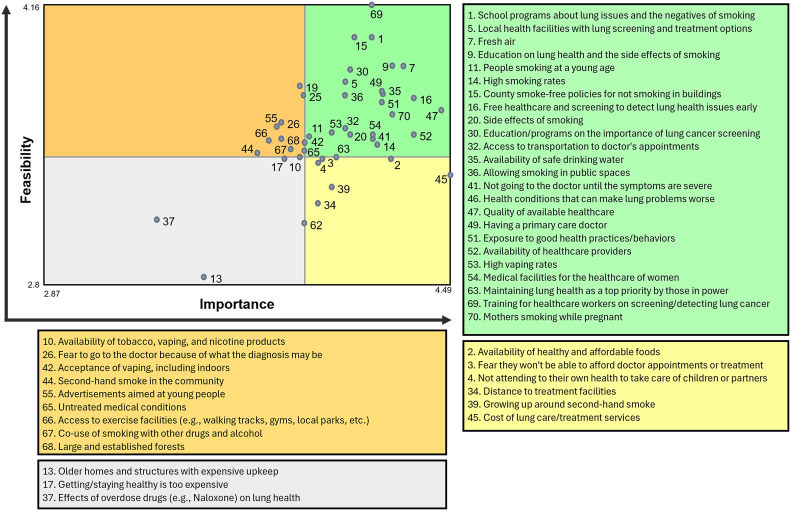
Go-Zone Plot for Top Rated Clusters (1, 2, 3, 6, and 7) With Items in Each Zone.

Figure 2 shows the comparison of the average cluster ratings across both rating scales, which were highly correlated (r = 0.95). In the interpretation discussion sessions, we grouped the clusters into three overarching areas: community-level (clusters 1 and 7), healthcare (clusters 2 and 3), and tobacco-related factors (cluster 6). These five clusters had the highest average cluster ratings across both rating scales, indicating that items within these clusters may be of particular importance and/or feasible to address. Figure 3 depicts the Go-Zone bi-variate plot for these top five rated clusters, with the items highly rated across scales in the green “go-zone” area.

### Focus Areas

#### Community-Level Factors

Items commonly raised by participants at the community-level focused on policy and educational factors. For example, all three groups pointed out the importance of items *15 (County smoke-free policies for not smoking in buildings)* and *1 (School programs about lung issues and the negatives of smoking)*. Participants in two of the groups also raised items *63 (Maintaining lung health as a top priority by those in power)* and *30 (Education/programs on the importance of lung cancer screening)*. Regarding smoke-free policies, one participant explained:

I think the smoke-free policies. I don’t know which counties have them. Sometimes when I’m travelling, I’m still shocked to see it [people smoking] when I’m in a restaurant or things like that. Because in the communities I’m in, that’s not allowed. So, I think focusing on those places where there’s still smoking allowed in county and city buildings, outside the doors of the hospitals, things like that are really important.

Participants further discussed how the implementation and continued enforcement of these policies requires the support and influence of those in power, many of whom see local politicians as putting “their efforts into things that are so stupid that just don’t amount to a hill of beans compared to death or living conditions or bad water issues - those should be priority things.” Discussions of education tended to focus on reaching younger individuals, women during pregnancy, and those eligible for lung cancer screening. Participants described a general lack of awareness in areas such as vaping risks and the lung cancer screening process. A participant stated: “There’s so many people, women especially, in my immediate social group that’s their defense [for why they vape]: ‘You don’t know there’s something wrong with this.’”

#### Healthcare Factors

Within the healthcare area, only one item consistently rose to the top of the discussions: *41 (Not going to the doctor until the symptoms are severe)*. One participant described the complex of healthcare seeking decision-making, particularly for a health issues like lung cancer which generates fear and is highly present in these communities:

If I know too much, I have all of these things going through my mind, and it scares me. And, do I really want to know the answer? Even though you really need to go to the doctor, you’re scared of what you might find. And that may be a reason why some women might not go and get the care that they need. Sometimes it just comes down to, I’ve got to bite this bullet and go get this checked out. I think that’s what it is… the symptoms outweigh the fear, but it can truly be too late then.

Additionally, practical considerations also played a key role in deciding to seek healthcare. For example, participants in various groups raised items *5 (Local health facilities with lung screening and treatment options)*, *34 (Distance to treatment facilities)*, and *16 (Free healthcare and screening to detect lung health issues early)*, emphasizing the role access, quality, and affordability of care continue to play in deciding to seek healthcare. Participants also discussed the training of the providers in their local communities, including item *16 (Training for health care workers on screening and detecting lung cancer)*. This item was often raised in connection with lung cancer screening; the majority of our participants had never heard of this screening, as one participant shares: “Do I have to ask for it? I mean I go to my doctor annually, but they’ve never suggested I have lung screening.” One participant who had experience with lung cancer screening elaborated on frustrations with current guidelines: “I wish it [lung cancer screening] weren’t just for people who smoke… I was raised with smokers and have been caretaker of two people with lung cancer. I have practically begged my physician to get screened.”

#### Tobacco Use Factors

Participants across groups commonly discussed generational variation in both the exposure and use of differing tobacco and nicotine products. All groups raised the importance of items *53 (High vaping rates)* and *42 (Acceptance of vaping, including indoors)*. Youth smoking also remained a concern, with items *11 (People smoking at a young age)* and *55 (Advertisements aimed at young people)* often arising in the discussions. The pervasiveness of advertisements regularly arose in conversation, as one participant stated:

Smoking is promoted in a lot of places. If you go to the gas station, there’s a lot of signs that say buy cigarettes, buy vape, buy this, cheap price… instead, you know, put anti-smoking campaign ads up, these are your lungs when you smoke, and this is how it affects the children in your car, instead of promoting it the way it is now.

Likewise, participants frequently mentioned that pregnancy is an ideal time for intervention, such as item *70 (Mothers smoking while pregnant)*. One participant summarizes:

The two groups I would focus on are the mothers and the teenagers, and I suppose the teenagers are mostly vaping right now. So, if you can keep them from starting smoking… I suppose if you’re pregnant you have a different attitude about health all together and if you can get to the middle schools and high schools when kids start vaping.

Finally, for older individuals, participants most often cited item *39 (Growing up around second-hand smoke)* as a key factor in continued generational smoking.

### Potential Intervention Strategies

At the end of each discussion group, we asked participants to highlight the most important area for future lung cancer prevention interventions and any related strategies. This conversation resulted in the identification of three key areas: 1) educational campaigns, particularly directed toward youth, mothers, and those eligible for lung cancer screening, focused on vaping, environmental risks, and screening resources; 2) policy, specifically ways to build a public movement around smoke-free laws, inclusion of vaping into existing policies, and advertisement bans; and 3) free and accessible lung cancer screening, including means to reduce barriers and meet people where they live.

Participants advocating for educational campaigns raised a number of strategies, including working with local organizations, Cooperative Extension Service offices, and recovery programs to create and share educational materials. Participants suggested highlighting personal stories and experiences within any developed educational materials and to consider generational differences (i.e., how the needs and tobacco use patterns among younger women may differ from older women in the community). A participant described how “word of mouth [here] can either hurt you or help you from somebody that was actually screened [or treated]… they tell someone else… and then the community knows,” highlighting the power of personal stories.

The conversation around policy solutions had fewer concrete suggestions but largely focused on ways to engage local community leaders who shape the norms and practices in the area. As one participant elaborates: “In [my town], there was smoking everywhere, and we had a doctor who really took this on as a cause, and eventually, [the town] adopted a no smoking policy, but it took somebody in power to make that happen.” Finally, participants suggested approaches including mobile screening units, expanding insurance coverage (or awareness of existing coverage), and utilizing non-physician, local community health workers to connect participants with screening opportunities. One participant shared:

People have different ideas about what lung cancer screening is all about… so maybe if we could get some non-professional healthcare workers. Maybe just people in the neighborhood who would know about screening and could be resource people to go to. I think that would be a really important thing.

## Discussion

### Principal Findings

Our results indicated that a focus on community-level awareness and change, including policy efforts and educational campaigns may reduce risk and increase prevention for lung cancer among women in Appalachia. Most participants seemed aware that KY does not have a state-wide smoke-free law, resulting in a patchwork of enacted laws at the county or municipal level with few laws currently present in Appalachia areas of the state.^9,38^ This inconsistency created confusion among women in our study, who wondered why such laws have not been enacted in all communities if they are effective at reducing exposure to tobacco smoke. Building awareness of the evidence to support smoke-free legislation among women, who often have a strong interest in reducing second-hand smoke exposure for themselves and their families, may create an opportunity for building momentum to advocate for change. Notably, although our participants seemed to be aware that environmental factors play a role in lung health, few raised radon specifically as a known risk factor for lung cancer.^14,15^ With the amount of time many women in Appalachian communities spend at home and the high levels of radon present in the region,^14^ a focus on building awareness as well as expanding programs to support radon testing and mitigation represent another important area for continued focus.

Interestingly, participants did not focus their discussion on tobacco cessation or on those who already smoke in their communities, which may reflect a perception that many efforts are already underway^39^ or a lack of belief that much change can be made. Instead, our participants focused on issues that affect youth^40,41^ and those during pregnancy^42,43^ as earlier time points in the life course to build awareness and behavioral change to reduce future lung cancer risk. In particular, the topic of vaping arose throughout our conversations, including how it fits into what smoke-free laws do exist and as a way to reach youth who may later expand to other nicotine or tobacco products.^44^ Likewise, our participants advocated for reducing commercial advertisements and increasing regulatory efforts to reduce the visual presence of tobacco and nicotine products in the community. These findings suggest women in Appalachian communities place a priority on stopping tobacco use before it starts as well as reducing harms in the community (e.g., second-hand smoke, advertisements) as a way to shift social norms.

Participants also highlighted the importance of the awareness and implementation of lung cancer screening in their communities. As with radon, few participants expressed knowledge of lung cancer screening or related guidelines,^45^ with those familiar expressing frustrations with the guideline focus on current smokers. Although guidelines intentionally focus on those most at risk,^46^ many of the women we spoke with have lived with individuals who smoke most of their lives with some serving as caregivers for those with lung cancer. Future research into updating guidelines should consider ways to measure second-hand smoke exposure as a potential addition to risk assessment and screening eligibility. Such an addition may be a way to reach non-smoking women who are at greater lung cancer risk. The discussion of strategies to increase lung cancer screening largely repeated similar themes among studies of other cancer screening sites,^47,48^ including an emphasis on non-physician supports, such as a community health workers, and ways to meet people where they are (e.g., mobile clinics, faith-based or other community locations). Continued use of peers as health educators for supporting behavior change may capitalize on the strong word of mouth networks in many of these communities to reduce risk and increase lung cancer prevention among Appalachian women.

Our findings support multilevel interventions for lung cancer prevention, including improving awareness, local policy, and screening access for Appalachian KY women. This research contributes novel understanding of local and gender-specific barriers and informs future Appalachian lung cancer prevention studies. Women in these communities desire new strategies to address non-smokers at risk for lung cancer (i.e., due to second-hand smoke or radon exposure) and more tailored existing tobacco cessation and lung risk reduction strategies that meet their needs. Incident cases of lung cancer among women in the US are projected to exceed those of men as of 2023, indicating current strategies, most of which were developed when male tobacco use was much more common than female tobacco use, may not work as well for this population. Future research should include an exploration of current evidence-based practices to reduce risk or prevent lung cancer among women, with particular focus among those in rural and Appalachian communities, and determine what adaptations or specific strategies would be most effective to prevent lung cancer among this population.

### Limitations

This study has a few notable limitations. Although we utilized several recruitment strategies to improve diversity of the sample, our participants are more educated than the general population of Appalachian KY. However, we did succeed in recruiting a relatively diverse sample in terms of race and ethnicity and in reaching women in 34 different counties, increasing the variety of thought and perceptions present in our data. Additionally, by collecting data online, we may have biased our sample towards those who are more likely to participate due to access to or familiarity with technology. In previous studies,^36^ we found high levels of participation in online studies among individuals in Appalachian communities, with few declining due to the technology utilized. All of our study components could be completed using mobile devices, rather than requiring a computer or broadband connection, increasing the ability to participate. Additionally, we ensured all of the Groupwisdom activities remained open for several weeks to decrease participant burden, allowing individuals to log-in and contribute at their own pace We also experienced high correlations between our rating scales; while it is common for importance rating scales to lack variability, we unexpectedly found a high correlation between the importance and feasibility item ratings, which limits our ability to identify areas uniquely important or feasible. However, the correlation also indicates high levels of agreement among participants for future areas of focus. Additionally, we experienced a lack of variability in ratings by demographics as well, limiting our ability to draw comparisons between participants by characteristics. Finally, our sample size is more in-line with qualitative studies and not capable of producing generalizable results; however, the goal of concept mapping is to create group consensus from heterogeneous perspectives. Our sample size of 71 is on the larger side for concept mapping studies and appropriate for our analyses.

**“So What?”**

**What is Already Known on This Topic?**

Lung cancer is the leading cause of cancer-related death for US women, including the Appalachian region, with decreases among women lagging behind those of men. Despite high rates, we currently lack published research on lung cancer risk and prevention among Appalachian women, indicating the need for novel primary and secondary prevention strategies.

**What Does This Article Add?**

Using concept mapping, a participatory mixed method, our participants listed 70 perceived barriers and facilitators affecting lung cancer prevention grouped in 8 thematic clusters, including community-level, healthcare, and tobacco-related factors. Our findings indicate three potential intervention areas: educational campaigns, policy, access to lung cancer screening.

**What are the Implications for Health Promotion Practice or Research?**

Changing trends in lung cancer incidence, with annual cases among women now exceeding men in the US, indicate the need for new prevention strategies. Our findings provide a novel exploration of barriers and facilitators to lung cancer prevention for Appalachian women and describes areas for continued focus within research and intervention development.

## Supplemental Material

Supplemental Material - Lung Cancer Prevention Among Appalachian Kentucky Women: A Community-Engaged Mixed Method Study Supplemental Material for Lung Cancer Prevention Among Appalachian Kentucky Women: A Community-Engaged Mixed Method Study by Jessica R. Thompson, Nancy E. Schoenberg, and Pamela C. Hull in American Journal of Health Promotion

## Data Availability

The datasets generated during and/or analyzed during the current study are available from the corresponding author on reasonable request.*
