# Supplementary material for: Lung Cancer Prevention Among Appalachian Kentucky Women: A Community-Engaged Mixed Method Study
Source: Am J Health Promot. 2025 May 27;39(8):1164–74. doi: 10.1177/08901171251346607 (PMC12476475; doi:10.1177/08901171251346607)
Supplement: Supplemental Material - Lung Cancer Prevention Among Appalachian Kentucky Women: A Community-Engaged Mixed Method Study [file sj-pdf-1-ahp-10.1177_08901171251346607.pdf]

Supplemental Table 1. Full List of Brainstorming Items in Numerical Order

#	Item
1	School programs about lung issues and the negatives of smoking
2	Availability of healthy and affordable foods
3	Fear they won't be able to afford doctor appointments or treatment
4	Not attending to their own health to take care of children or partners
5	Local health facilities with lung screening and treatment options
6	Stress women experience in their lives
7	Fresh air
8	Attitude of "it is what it is" when it comes to health
9	Education on lung health and the side effects of smoking
10	Availability of tobacco, vaping, and nicotine products
11	People smoking at a young age
12	Alcohol use/excess drinking
13	Older homes and structures with expensive upkeep
14	High smoking rates
15	County smoke-free policies for not smoking in buildings
16	Free healthcare and screening to detect lung health issues early
17	Getting/staying healthy is too expensive
18	Air fresheners
19	Use of prescription inhalers and medicines to improve lung health
20	Side effects of smoking
21	Not caring what others think/social pressures about quitting smoking
22	Use of smoking among people in recovery/treatment from drug abuse and alcoholism
23	Acceptance and normalization of smoking
24	Climate conditions
25	Ability to get screened at a younger age
26	Fear to go to the doctor because of what the diagnosis may be
27	Chemicals/pesticides being used in local communities
28	Working in places that could be risky to lung health
29	Pollution from vehicles (e.g., cars, diesel trucks, farm machinery)
30	Education/programs on the importance of lung cancer screening
31	Environmental toxins in the home like plastics and radon gas
32	Access to transportation to doctor's appointments
33	Peer pressure
34	Distance to treatment facilities
35	Availability of safe drinking water
36	Allowing smoking in public spaces
37	Effects of overdose drugs (e.g., Naloxone)
38	High asthma rates
39	Growing up around second-hand smoke
40	Obesity
41	Not going to the doctor until the symptoms are severe
42	Acceptance of vaping, including indoors
43	Asbestos in houses and other buildings
44	Second-hand smoke in the community
45	Cost of lung care/treatment services
46	Health conditions that can make lung problems worse
47	Quality of available healthcare
48	Run-off from coal mines
49	Having a primary care doctor
50	Substance use
51	Exposure to good health practices/behaviors
52	Availability of healthcare providers
53	High vaping rates
54	Medical facilities for the healthcare of women
55	Advertisements aimed at young people

56	Growing up watching others, including women, in the family smoke
57	Pollution from manufacturing companies, plants, or factories
58	Pollen in allergy seasons (Spring and Fall)
59	Open burns of vegetation and garbage
60	Miners coming home covered in coal dust
61	Genetics
62	Low household income
63	Maintaining lung health as a top priority by those in power
64	Smoking as a way to socialize
65	Untreated medical conditions
66	Access to exercise facilities (e.g., walking tracks, gyms, local parks, etc.)
67	Co-use of smoking with other drugs and alcohol
68	Large and established forests
69	Training for health care workers on screening and detecting lung cancer
70	Mothers smoking while pregnant
